# A compound heterozygous *ADAMTS13* mutation causes congenital thrombotic thrombocytopenic purpura: a case report

**DOI:** 10.3389/fmed.2024.1525062

**Published:** 2025-01-08

**Authors:** Yezi Huang, Lixia Zhou, Yuan Song, Wanting Zou, Aiping Tang, Si Tao, Duozhuang Tang

**Affiliations:** ^1^Department of Hematology, The Second Affiliated Hospital of Nanchang University, Nanchang, China; ^2^Department of Oncology, The Second Affiliated Hospital of Nanchang University, Nanchang, China

**Keywords:** congenital thrombotic thrombocytopenic purpura, *ADAMTS13*, thrombotic microangiopathy, sC5b-9, case report

## Abstract

Congenital thrombotic thrombocytopenic purpura (cTTP) is a thrombotic microangiopathy (TMA) characterized by severe hereditary ADAMTS13 (a disintegrin and metalloproteinase with thrombospondin type 1 motifs 13) deficiency caused by *ADAMTS13* mutations. This rare autosomal recessive genetic disorder is often misdiagnosed as immune thrombocytopenia (ITP) or hemolytic uremic syndrome (HUS). Here, we report a 21-year-old male cTTP patient with a compound heterozygous *ADAMTS13* mutation. The patient was admitted for acute thrombocytopenia, with a 5-year history of chronic thrombocytopenia and 1 month of renal dysfunction. Initially diagnosed with ITP, he was treated with immunosuppressive therapy, including glucocorticoids and intravenous immunoglobulin, which provided temporary relief but failed to prevent recurrent thrombocytopenia. Ultimately, cTTP was confirmed by the low ADAMTS13 0% activity and two heterozygous variants (c.1335del and c.1045C > T) in the *ADAMTS13* gene, and the patient received prophylactic fresh-frozen plasma (FFP) infusions every 2–3 weeks regularly. Interestingly, the patient also exhibited elevated sC5b-9 levels during the acute phase, necessitating differentiation from HUS. This report highlights a cTTP caused by a compound heterozygous *ADAMTS13* mutation, although its pathogenesis requires further investigation. Given the atypical clinical manifestations of cTTP, it is necessary to conduct ADAMTS13 activity and even genetic testing in patients with recurrent thrombocytopenia and end-organ damage.

## 1 Introduction

Thrombotic thrombocytopenic purpura (TTP) is a thrombotic microangiopathy (TMA) resulting from hereditary or immune-mediated deficiency of the enzyme ADAMTS13 (a disintegrin and metalloproteinase with thrombospondin type 1 motifs, member 13) activity, leading to impaired cleavage of high-molecular-weight von Willebrand factor (HMW-VWF) multimers, with clinical manifestations of severe thrombocytopenia, microangiopathic hemolysis, and thrombosis-induced multiorgan functional impairment (1). cTTP is caused by *ADAMTS13* mutations resulting in reduced synthesis or increased clearance induced by the instability structure of ADAMTS13, representing only 5% of TTP patients (2). However, even among patients with the same variant, disease onset and the clinical course exhibit considerable heterogeneity. Therefore, the pathogenesis of this disease warrants further investigation. Here, we present a new compound heterozygous *ADAMTS13* mutation, which has not been reported in a Chinese male adult.

## 2 Case description

**Figure 1:**
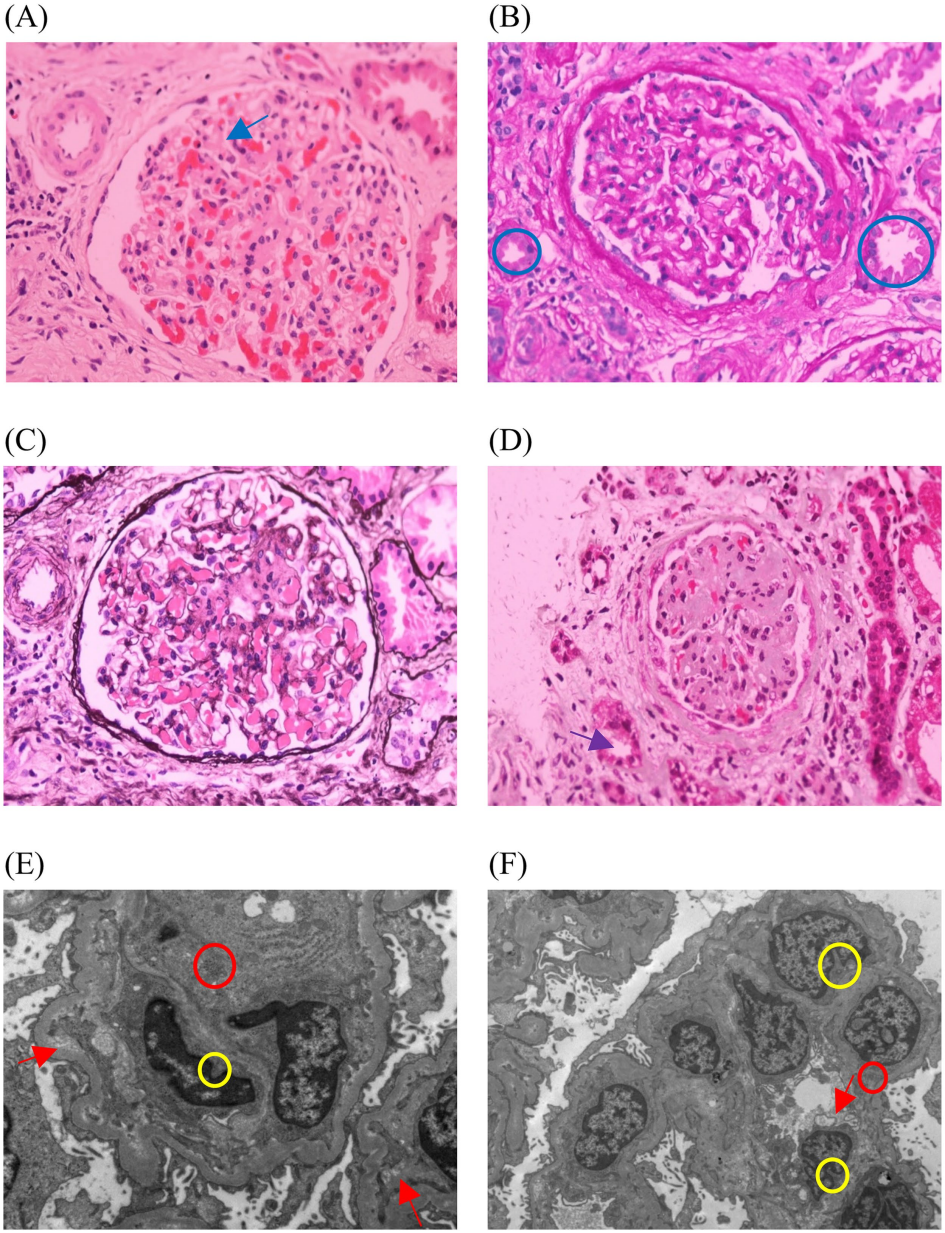
Images of the patient’s renal needle biopsy. **(A)** The blue arrow shows glomerulosclerosis, with 8 out of 15 glomeruli showing sclerosis, and no segmental glomerulosclerosis (HE). **(B)** The blue circles show protein casts (PAS). **(C)** The glomerular basement membrane shows no thickening, with no double-track (PASM). **(D)** The purple arrow shows the atrophic change in the renal tubules, with approximately 40% of the tubular area showing atrophy (Masson). **(E,F)** The yellow circles show segmental mesangial matrix insertion into the glomerular basement membrane, forming a double-track structure. The red arrows show vacuolar degeneration of the renal tubular epithelial cells. The red circle shows focal infiltration of inflammatory cells in the renal interstitium. The foot processes exhibit segmental fusion changes. Both mesangial cells and the matrix show mild proliferation (EM).

The patient was a 21-year-old male who developed renal dysfunction and edema after experiencing 5 years of chronic moderate thrombocytopenia. He was first diagnosed with immune thrombocytopenia (ITP) at the age of 16 years for skin purpura and moderate thrombocytopenia at a local hospital. The patient had no other medical or family history. His parents were not consanguineous but had a history of bearing a male infant with nasolabial fold dysplasia who died during infancy. His sister is healthy. He did not receive any therapeutic interventions during the following 5 years, and his platelet number remained between 50 and 70 × 10^9^/L (normal range: 125–350 × 10^9^/L), with other blood cell line numbers remaining normal. At the age of 21 years, he presented with lower extremity edema and was subsequently hospitalized in the nephrology department. Biochemical tests revealed: total bilirubin (TBILI), 26.39 μmol/L (normal range: 0–23 μmol/L); unconjugated bilirubin (UCB), 22.03 μmol/L (normal range: 0–19 μmol/L); lactate dehydrogenase (LDH), 354.25 U/L (normal range: 120–250 U/L); and serum creatinine (Scr), 198.9 μmol/L (normal range: 57–97 μmol/L). Blood tests showed a platelet count of 34 × 10^9^/L and a hemoglobin (Hb) level of 144 g/L (normal range: 130–175 g/L). Renal biopsy revealed mild mesangial proliferative glomerulopathy with partial glomerulosclerosis (8/15) and moderate (40%) chronic tubulointerstitial injury (Figure 1). Following treatment with corticosteroids, the patient developed hypertension (140/100 mmHg), which was subsequently controlled to approximately 135/85 mmHg with triple antihypertensive therapy. One month later, he presented to the hematology department for severe thrombocytopenia. Subsequent tests showed platelets 9 × 10^9^/L, Hb 109 g/L, Scr 168.43 μmol/L, LDH 702 U/L, TBILI 47.44 μmol/L, and UCB 40.13 μmol/L. Examination of the marrow smear showed abnormalities in megakaryocytic lineage maturation and thrombocytopenia. The cranial CT scan shows no abnormalities. Given his history of chronic thrombocytopenia, the patient was diagnosed with ITP, and treated with platelet transfusions, dexamethasone, and intravenous immunoglobulin (IVIG). His platelet count improved to 90 × 10^9^/L within 1 week; however, thrombocytopenia and mild anemia recurred within 1 month. These treatments were repeated over the following 6 months.

**Figure 2:**
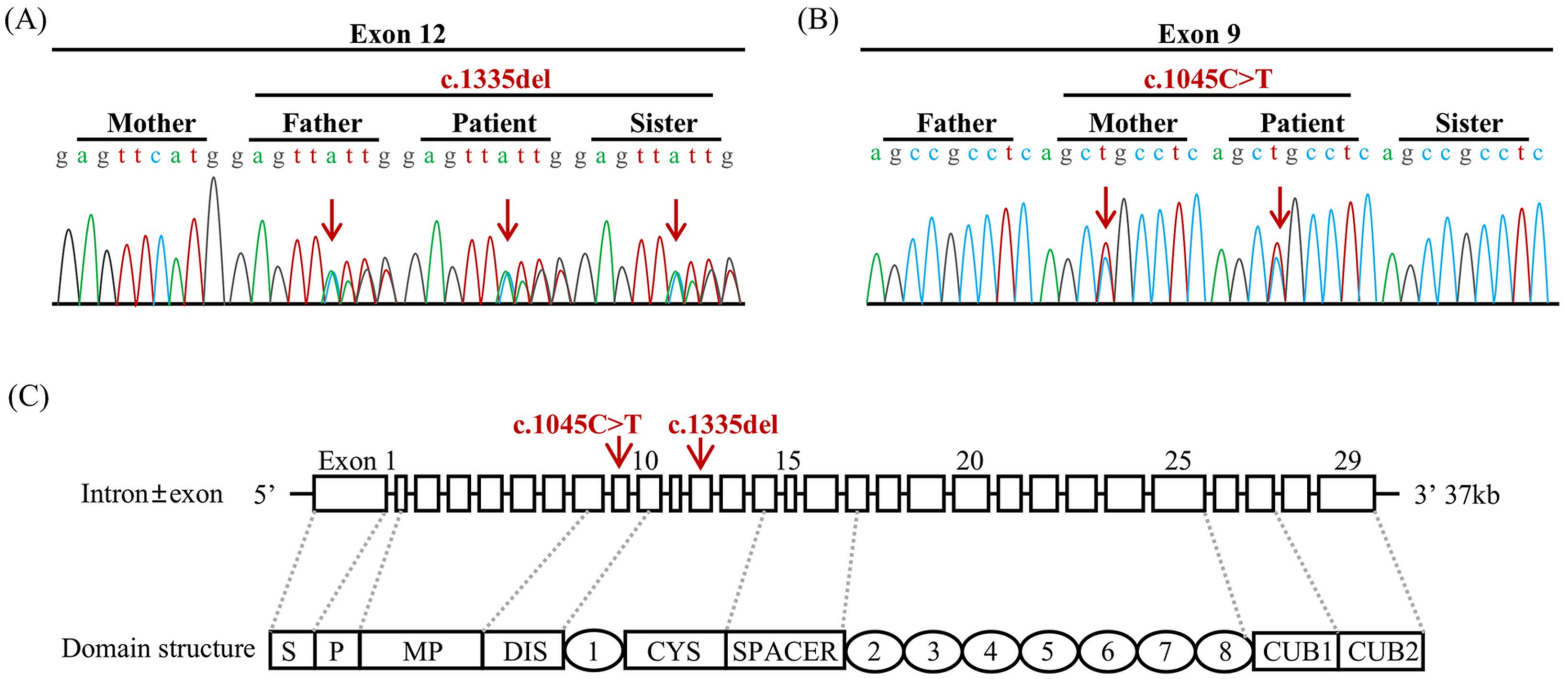
Sequencing of the mutation in the *ADAMTS13* gene and intron ± exon and domain structures of ADAMTS13. **(A)** The patient, along with his father and sister, carried the heterozygous variant c.1335delC (p. Phe445LeufsTer52). **(B)** The patient and his mother carried the heterozygous variant c.1045C > T (p.Arg349Cys). The red arrows indicate the base change in the DNA. **(C)** DAMTS13 is a metalloprotease that consists of a signal peptide domain (S), a pro-peptide domain (P), a metalloprotease domain (MP), a disintegrin domain (DIS), a series of thrombospondin type 1 (TSP 1), a cysteine-rich domain (CYS), and a spacer domain (Spacer). Additionally, the C-tail consists of seven more repeats of thrombospondin type 1 (TSP 2–8) and CUB domains (CUB1-2). The red arrows indicate the locations of the variants in the exon.

Considering the potential diagnoses of TTP or hemolytic uremic syndrome (HUS), further tests were conducted, including the gene mutation analysis for coagulopathy and hematologic malignancies, measurement of sC5b-9, complement factor I and complement factor H levels, and ADAMTS13 activity. The truth came to light, and the patient was diagnosed with cTTP, based on compound heterozygous variants (c.1335del and c.1045C > T) in the *ADAMTS13* gene, absence of ADAMTS13 inhibitors, and an ADAMTS13 activity level of 0%. Interestingly, the patient had an elevated sC5b-9 level of 754 ng/mL but no complement gene mutations were identified. Through Sanger sequencing, it was validated that the patient also carries germline heterozygous variants in the following genes: NM_015338.6 (*ASXL1*): c.1907C > T, p.Ala636Val; NM_001039396.2 (*MPEG1*): c.1108G > T, p.Glu370*; NM_014643.4 (*ZNF516*): c.3352C > G, p.Arg1118Glyfs*42; NM_015001.3 (*SPEN*): c.6719C > T, p.Pro2240Leu; NM_001164273.2 (*MGA*): c.7700G > C, p.Arg2567Thr; and NM_000038.6 (*APC*): c.5376 T > G, p.N1792K; thus, the patient’s health status warrants further follow-up and attention. Moreover, the family investigation revealed that the patient’s parents and sister also carried heterozygous *ADAMTS13* mutation (Figure 2), but had normal ADAMTS13 activities (Table 1) and were in good health.

**Table 1:** ADAMTS13 activities and inhibitor test results of the patient and his families.

	ADAMTS13 activities (%)	ADAMTS13 inhibitor (BU/mL)
Father	>10%	0
Mother	>10%	0
Patient	0	0
Sister	>10%	0

**Figure 3:**
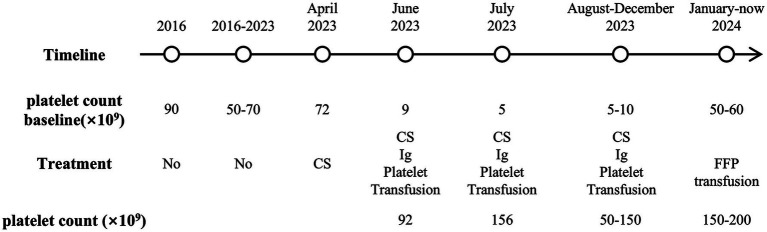
Timeline of the case. CS, corticosteroid; Ig, gamma globulin; FFP, fresh frozen plasma.

Upon confirmation of cTTP, the patient subsequently received regular prophylactic fresh frozen plasma (FFP) infusions of 5 mL/kg every 2–3 weeks, which have been ongoing for nearly 1 year. Platelet levels peaked approximately 1 week post-transfusion, but fell to the baseline after 2 weeks, fluctuating between 50 and 200 × 10^9^/L, with creatinine fluctuating between 160 and 180 μmol/L. The patient is currently in overall stable condition, with no edema or purpura, and his blood pressure is well controlled. Aside from chronic kidney insufficiency, there is no evidence of other ischemic end-organ damage. The timeline of this case is shown in Figure 3.

## 3 Discussion and conclusion

We report a patient who developed renal insufficiency and a sharp decline in platelet count after 5 years of chronic mild thrombocytopenia. The patient also exhibited elevated levels of sC5b9, but no complement gene mutations were detected. Ultimately, the patient was diagnosed with cTTP rather than HUS, based on ADAMTS13 activity being below 10%, the pathogenic *ADAMTS13* mutations, and the absence of ADAMTS13 inhibitors (3). The correlation between *ADAMTS13* genotype, immune status, and disease phenotype in cTTP warrants further investigation.

cTTP may be difficult to recognize before organ failure associated with microvascular thrombosis occurs. The classic “pentad” of TTP is rarely observed, making the diagnosis particularly challenging. Traditional distinction between TTP and HUS is based on the predominant neurological involvement and more severe thrombocytopenia in TTP and more severe acute renal injury in HUS. However, this distinction is often unclear as clinical presentations may overlap (4). Our patient initially presented to our hospital due to renal dysfunction. Zafrani et al. (5). reviewed the patients with TTP and found that AKI is present in more than half the patients, and half of those will have lasting renal effects. Active renal lesions in TMA include red blood cell fragmentation, endothelial swelling, glomerular or arteriolar occlusion, mesangiolysis, or glomerular microaneurysms, while chronic lesions include double contours in the glomerular basement membrane and intimal fibrosis of the arteries (6). Itami et al. (7) studied five cTTP patients, most of whom demonstrated chronic glomerular changes, including collapsed capillaries, global or segmental sclerosis, and duplication of basement membranes. Four cases also showed interstitial fibrosis/tubular atrophy. The kidney pathology in our patient also demonstrated findings consistent with chronic kidney injury. After regular plasma infusions, the patient showed a decrease in creatinine alongside an increase in platelet count, with a significant reduction in urine protein compared to before, which suggests that plasma infusion can help prevent further progression of end-organ damage, although the damage may be irreversible.

Our study identified compound heterozygous ADAMTS13 variants in this patient. The frameshift variant (c.1335del, Exon12), located in the cysteine-rich domain (Figure 2C), has been previously reported in two cTTP cases in China (8, 9). It may change ADAMTS13 structure significantly by altering amino acid synthesis from phenylalanine 445 to amino acid 52 (p.Phe445LeufsTer52). According to ACMG guidelines, this variant is classified as Likely Pathogenic (PVS1+ PM2_Supporting). The missense variant (c.1045C > T, Exon9), located in the disintegrin-like domain (Figure 3C), changes amino acid 349 from arginine to cysteine (p.Arg349Cys), inactivating the catalytic domain of ADAMTS13 and reducing its secretion or protein hydrolytic activity (10, 11). This variant has been detected in more than two cTTP patients and coexists in trans with pathogenic or likely pathogenic variants. It is classified as Uncertain (PM2_Supporting + PM3_Strong). The two variants have a very low frequency in the population database, and their combination has not been reported before. Lotta et al. (12) found that *ADAMTS13* mutations in the N-terminal domain are associated with lower enzyme activity and severe disease in an allele-dosage-dependent manner. Alwan et al. (13) reviewed a large cohort of cTTP cases and found that prespacer mutations are more likely to be associated with childhood onset. Hassenpflug et al. (10) investigated the German cTTP cohort and found that severe phenotypes are almost always caused by the combination of two mutations, which result in the production of completely inactive and/or severely altered proteins from both alleles. When both mutants showed residual activity, a mild phenotype was observed. It explains why there is no simple genotype/phenotype correlation for single mutations in compound heterozygous patients. Currently, establishing a genotype–phenotype correlation remains challenging.

In addition to mutations, the “second hit hypothesis” suggests that lack of ADAMTS13 activity alone is insufficient to cause TTP and that a combination of inflammatory factors, vascular endothelial damage, and aberrant complement activation triggers disease onset. In this case, the patient first presented with renal insufficiency as well as medically uncontrollable hypertension, followed by a dramatic thrombocytopenia. No obvious triggers were detected, such as infections, vaccinations, or patent ductus arteriosus in infancy (14, 15). Studies have shown that serum creatinine levels correlate positively with vWF (16). We hypothesize that the acute thrombocytopenia in this patient is partly associated with the impaired renal function that reduces the degradation of vWF, or endothelial damage aggravated by elevated toxin levels (e.g., creatinine) and blood pressure, which serves as a “second hit” that leads to excessive vWF–platelet interactions and subsequently platelet depletion. Additionally, elevated sC5b-9 complement (754 ng/mL) was observed during the patient’s acute exacerbation. Many studies have emphasized the role of complement activation in the pathogenesis of TTP. When ADAMTS13 is absent, endothelial ULVWF chains, which are inefficiently cleared, may serve as templates for assembling complement components, leading to local complement overactivation and the formation of terminal C5b-9 complexes, thus causing endothelial injury and thrombosis (17). Cugno et al. (18) recently found that plasma sC5b-9 levels were significantly elevated in TTP patients with renal insufficiency and that serum creatinine levels were positively correlated with sC5b-9 levels during the acute phase of TTP, which is consistent with our report. In addition, the reported effectiveness of the C5 inhibitor eculizumab in cTTP may provide insights into new therapeutic approaches for TTP (19). However, the role of complement activation as a driving event or merely a secondary finding in many other TTP conditions remains unclear, which warrants further investigation.

In conclusion, the study reported a case of cTTP associated with a compound heterozygous *ADAMTS13* mutation and elevated sC5b-9 levels. The report has some limitations. For instance, corticosteroids were used before testing for antibodies, which may have caused false-negative results. We recommend that ADAMTS13 activity, inhibitors, and even genetic mutation testing should be conducted before starting treatment for patients with recurrent thrombocytopenia and organ damage. As the potential link between cTTP and its phenotype has not yet been established, it is hoped that this compound heterozygous mutation may provide a reference for the clinical phenotype of cTTP patients.

## Data Availability

The original contributions presented in the study are included in the article, further inquiries can be directed to the corresponding author.
